# Economic Inequality, Life Expectancy, and Interpersonal Violence in London Neighborhoods

**DOI:** 10.1177/08862605241271379

**Published:** 2024-08-24

**Authors:** Jaye Lee McLaughlin, Nicholas Pound

**Affiliations:** 1 Centre for Culture & Evolution and Division of Psychology, Brunel University London, Uxbridge, UK

**Keywords:** economic inequality, life expectancy, violent crime, assault, community violence

## Abstract

Positive associations between levels of socioeconomic inequality and homicide rates have been reported at various geographical levels (e.g., between countries, states, cities, and neighborhoods within a city). However, the extent to which inequality predicts levels of non-lethal violence has been less frequently studied. The present study was conducted to investigate the association between socioeconomic inequality and levels of non-lethal interpersonal violence across neighborhoods of London during the period 2010 to 2012, using two independent data sources: Metropolitan Police service recorded violent crime and London Ambulance Service recorded assaults. Mean income per person and local life expectancy were included as additional predictors. Following exclusions due to census boundary changes, across 533 London wards, there were positive bivariate associations between both violence measures and a measure of inequality between neighborhoods (census lower layer super output areas [LSOAs]) within a ward. Moreover, there were negative bivariate associations between violence rates and both ward mean income and life expectancy measures for males and females. However, in a regression analysis only inequality and male life expectancy were consistent predictors of rates of interpersonal violence across outcome measures. The results of the present study provide further evidence of an association between levels of economic inequality and rates of interpersonal violence. The findings, for variation in rates of non-lethal violence across small geographical areas (neighborhoods), build on previous research that has mostly focused on rates of lethal violence and has tended to use aggregate measures across larger geographical areas.

## Introduction

High levels of socioeconomic inequality are associated with a range of negative outcomes for societies and communities—including poorer physical and mental health for individuals, lower levels of interpersonal trust, and higher crime rates (Wilkinson & Pickett, 2007, 2009). The association between inequality and violent crime (Fajnzylber et al., 2002; Wilkinson, 2004) in particular, is an issue of great concern to the public and policymakers, as interpersonal violence is a major cause of morbidity and mortality among young people (World Health Organization, 2010).

The link between inequality and violence has been most widely studied for an extreme manifestation of interpersonal conflict—homicide. Criminology research has shown that levels of socioeconomic inequality are correlated with homicide rates across countries (Elgar & Aitken, 2011; Daly & Krupp, 2022), and within the U.S. positive associations between levels of inequality and homicide rates have been observed across states (Kennedy et al., 1996), counties (Rowhani-Rahbar et al., 2019), cities (Blau & Blau, 1982) and neighborhoods within a city (Wilson & Daly, 1997). Similarly, across cities in eight Latin American countries, those with higher levels of income inequality have higher homicide rates (de Lima Friche et al., 2023); while within Rio de Janeiro inequality indicators predicted variation in homicide rates between neighborhoods whereas general measures of poverty did not (Szwarcwald et al., 1999).

As inequality often covaries with other factors that may predict levels of violence (e.g., levels of absolute poverty) it can be difficult to disentangle its particular contribution. However, in a number of cross-national studies, when considered alongside a range of other predictors in regression models, socioeconomic inequality has emerged as the most consistent, and frequently the strongest predictor of homicide rates (Daly, 2016, 2023). Moreover, in a within-country analysis across Canadian provinces (where unusually the most inequitable provinces are also the most affluent) income inequality, but not mean income, predicted homicide rates (Daly et al., 2001).

Both sociological strain theory (Rennó Santos et al., 2018) and an evolutionary psychological perspective (Daly, 2016) can provide complementary explanations for these observed associations—based around the idea that experiences of relative deprivation will tend to increase the likelihood of individuals resorting to interpersonal violence when they come into conflict. Strain theory predicts that inequitable distribution of resources may lead the relatively deprived to feel alienated from society (Kelly, 2000) which may be a proximate factor motivating crime. Moreover, an evolutionary perspective can shed light on the ultimate origins of such psychological responses. Briefly, such an approach suggests that when resources are distributed inequitably there will be increased competition for access to those resources, and under these circumstances individuals who are relatively deprived will feel they have little to lose from adopting more risky modes of social competition, including violence (Daly, 2016; Wilson & Daly, 1997; Wilson et al., 2009). At a proximate psychological level this will tend to manifest itself in the form of greater proclivity for risk taking and a tendency to prefer smaller immediate gains over larger delayed rewards (i.e., to display steep future discounting^1^) in inequitable environments (Mishra et al., 2015; Payne et al., 2017).

There are some inconsistencies in the literature on associations between inequality and homicide. However, it is important to bear in mind that homicides arise primarily from violent altercations, most of which do not have fatal outcomes, and homicides can thus be considered the “tip of an iceberg” and probable indicator of the incidence of violence interpersonal conflicts in general (Daly & Wilson, 1988). Consequently, to shed further light on the associations between experiences of socioeconomic inequality and interpersonal violence, it can be informative to study non-lethal manifestations of conflict (e.g., assaults). Cross-national comparisons in this area are problematic due to different policing and crime-recording practices. Moreover, inequality at a more local level may be a more important than societal level inequalities as a factor contributing to levels of interpersonal conflict for a number of reasons (Krupp & Cook, 2018). Most competitive interactions between individuals are highly local in nature, arising for example when people compete over the same resources, social status, and potentially sexual partners (Daly & Krupp, 2022). Moreover, when competition is local this can amplify the effects of inequality on risk-taking (Krupp & Cook, 2018). Consequently, examining associations between inequality and violence across smaller geographical areas may help resolve some outstanding issues.

For some locales detailed datasets are available—where data on assaults has been published for small neighborhood areas, collected by a single public body (e.g., a police force) across a region using consistent reporting standards. One such locale is London, in the United Kingdom, where the Metropolitan Police service and the London Ambulance Service (LAS) have collected and published data on numbers of violent crimes and assaults across the city, at neighborhood level and in a relatively consistent manner over a period of years. Accordingly, the present study was carried out to examine whether there was a positive association between levels of income inequality and rates of police recorded violent crime and ambulance service recorded assaults during the period 2010 to 2012, that is, around the time of the 2011 census, a period for which good estimates of relevant socioeconomic and demographic variables are available using the same neighbored boundaries. Two independent measures of the incidence of interpersonal violence were used, as using ambulance service data on incidents of interpersonal violence can provide important additional information on violent interactions that may not be recorded by the police (Sutherland et al., 2021). The largely independent nature of the data sources means they assess levels of interpersonal conflict and violence in slightly different ways. For example, the LAS data may include assaults with injury for which there was no police involvement, while the police data may include interactions involving violence where no injury warranting ambulance service involvement occurred.

As in Wilson & Daly’s (1997) classic study demonstrating the association between economic inequality and homicide at neighborhood level, we have also included local life expectancy as a potential demographic predictor of variation in the incidence of interpersonal violence. In that study, they found a strong negative association between life expectancy (excluding homicide as a cause of death) and homicide rates across Chicago neighborhoods. They argued that such a pattern may arise due to reduced expectations of longevity causing individuals to discount the future more steeply, and to be more willing to engage in risky behaviors in the context of social competition. Consistent with this idea, more recent research has found that early environmental unpredictability and lower perceived life expectancy predict self-reported antisocial behavior (Dickerson & Quas, 2021). Researchers in some fields have tended to refer to steep future discounting in somewhat pejorative terms (e.g., “shortsightedness” or “inability to delay gratification”). However, such discounting may reflect functional/adaptive responses of individuals to social/environmental conditions that mean they are less likely to survive to derive benefits from delayed rewards (Daly & Wilson, 2005; Wilson et al., 2009).

## Methods

### Police Service Recorded Violent Crime

Data on the number of “violence against the person” offenses collated by London’s Metropolitan Police Service and published by the London Datastore^2^ were used to calculate the monthly incidence of violent crimes recorded by the police during the corresponding period (April 2010 to March 2012). The Metropolitan Police dataset covers “notifiable offenses” (i.e., crimes that all police forces in England and Wales are required to report to the Home Office) and provides monthly counts for each offense type for different geographical areas. Recorded counts of “Violent crime” offences were used to calculate incidence rates (offences per month per 1,000 population) for each of London’s 624 electoral wards in London (excluding the City of London which is covered by a different police force—the City of London Police). The “Violent Crime” (“violence against the person”) data “includes a range of offences from minor offences such as harassment and common assault, to serious offences such as murder, actual bodily harm and grievous bodily harm.”^3^

### Ambulance Service Recorded Assaults

Data published by the LAS^4^ were used to calculate the monthly incidence rate of assaults occurring in each of the 624 electoral wards in London (excluding the City of London). This dataset includes counts, by geographical area of all callouts for which, paramedics or other ambulance staff, have recorded retrospectively that they believe an assault took place. The published data do not indicate sex of offender; however, separate totals are provided for “Assaults on Women.” Consequently, for each ward, incidence rates (incidents per month per 1,000 population) were calculated for Total Assaults and Assaults on Women for the period April 2010 to March 2012.

### Socioeconomic and Demographic Data

Population data used to calculate the assault / violent crime incidence rates for each ward were derived from the 2011 U.K. Census. Life expectancy data for each ward were UK Office for National Statistics (ONS) rolling 5-year combined life expectancy estimates for the period 2009 to 2013 published in 2016 but based on pre-2011 boundaries. To avoid circularity, Wilson & Daly (1997) removed homicide as a cause of death from the life expectancy statistics they used to predict homicide rates in Chicago neighborhoods. However, we have not done this here as the dependent measures being predicted are measure of the incidence of primarily non-fatal assaults rather than homicides. Moreover, homicide rates are very low in London (<2.0 per 100,000 per year, 2010–2012) (Morgan et al., 2020) compared to Chicago (>20 per 100,000 per year, 1988–1993) (Block & Christakos, 1995) during the respective study periods and are therefore not a major contributor to life expectancy statistics in London reducing the possibility of circularity. There were approximately 250,000 violent crimes in the police dataset for the study period (April 2010 to March 2012) and this would have included the approximately 250 homicides that took place in London during that time. So approximately 99.9% of the recorded violent crimes in our analyses are not homicides.

To quantify levels of affluence and inequality within each of the electoral wards, demographic data from the 2011 U.K. Census were used along with modeled estimates of household income published by the Office of National Statistics for each of the lower super output areas (LSOAs) in the London boroughs (excluding the City of London). Wards in London typically contain six to nine LSOAs (mean 7.63; *SD* 1.29). Average annual income per person for each LSOA was calculated as average household income × number of households/total LSOA population. Average annual income per person for each ward was calculated in a similar manner. This approach was taken as household income alone would be confounded by household composition. Moreover, we did not have access to relevant data for 2010 to 2012 at LSOA level that would allow us to use, or calculate, household equivalized income using OECD equivalization methods.

### Inequality

As in many previous studies of associations between inequality and crime (Daly et al., 2001, de Lima Friche et al., 2023; Szwarcwald et al., 1999) we have used Gini coefficients as a measure of how inequitably resources are distributed between units (e.g., individuals or households). In technical terms, the Gini coefficient (which can vary between 0.0 and 1.0) is based on the Lorenz curve of the income distribution which is constructed by ranking units of analysis (e.g., individuals or households) and then creating a plot of cumulative income share against the cumulative number of individuals or households (see Supplemental Figure 5 for an example). The Gini coefficient is the area between the Lorenz curve, and a straight line representing complete equality (e.g., see Office for National Statistics, 2022, April 26, for a further explanation). When incomes are completely equitable, that is all units in an analysis have identical incomes, then the Gini coefficient will equal zero. As inequality in incomes increases, the Gini coefficient will increase, reaching a maximum 1.0 in the hypothetical situation where all income is received by one unit only.

**Figure 1.**
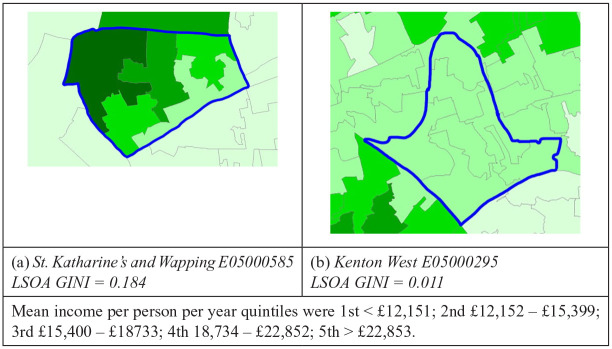
Example of wards with high (a) and low (b) LSOA Gini. LSOAs shaded by quintiles for mean income per person per year. *Note.* LSOA = lower super output area.

For the present study, individual level income data were not available, so we have used small geographical areas (LSOAs) as the units of analysis—and computed Gini coefficients as a measure of inequality between these smaller geographical units within each ward. A Gini coefficient was calculated for each ward (“Ward LSOA Gini”), to quantify the degree of inequality in income per person between the LSOAs that comprise the ward (i.e., each LSOA was treated as an “individual” in the Gini calculations, see Figure 1 for examples). As the average number of LSOAs per ward is only 7.63, and Gini coefficients can be biased downwards with small sample sizes (Deltas, 2003) an alternative adjusted formula for Gini with small sample sizes was used (Deaton, 1997; Deltas, 2003).

$${\hat{G}}_{n}\;=\;\frac{1}{\bar{y}n(n-1)}\sum_{i>j}\sum_{j}\left|\;y_{i}-\;y_{j}\right|$$

### Data Exclusions

In 2011, the boundaries were changed for 207 of London’s 4,835 LSOAs (excluding the City of London), across 90 of the 624 electoral wards. To account for changes in population size; some of them were merged, and some were split. For data available covering the period 2010 to 2012 the LAS data used the pre-2011 boundaries, while the ONS and Metropolitan Police Service data used the post-2011 boundaries. These boundary changes meant it was not possible to match economic data and assault data for 90 of the electoral wards. Consequently, all wards containing LSOAs with boundary changes were excluded from the analysis so that LAS and Metropolitan Police Service data could be considered simultaneously in the same analyses. This left 534 wards eligible for inclusion. In addition, two outliers in terms of crime statistics were excluded from the analyses. St James’s (E05000644) and West End (E05000649) wards had per capita assault and violent crime rates 8.8 to 14.7 standard deviations above the mean, respectively, which was probably due to their high density of retail, commercial and tourist activity without commensurately high residential populations. However, West End (E05000649) was already excluded due to LSOA changes so only 1 ward (St James’s) was excluded as an outlier, leaving 533 wards in the analysis.

## Results

For the 533 wards included in the analyses, as shown in Table 1, one-sample Kolmogorov-Smirnov (K-S) tests showed that all ward level variables (except Female Life Expectancy) exhibited significant departures from normality with most exhibiting moderate-to-high positive skew, and several variables also exhibiting substantial kurtosis. Accordingly, log transformed values of all variables have been used in the analyses presented here. Figures showing quintiles for Gini, income, violence crime, and ambulance service recorded assaults for wards across London are provided in the Supplemental Material.

**Table 1.** Descriptive Statistics and Tests for Normality for Ward Level (*n* = 533) Study Variables.

Ward Level Variable	Mean	Min	Max	*SD*	Skew	Kurtosis	K-S	*p*
Mean annual income per person (2010/2011)	£20,455	£9,022	£69,116	£8,941	2.50	8.42	0.15	<.001
Ward Gini (2010/2011)	0.06	0.01	0.20	0.03	1.17	1.85	0.10	<.001
Violent crimes per month per 1,000 (April 2010 to March 2012)	1.48	0.36	5.68	0.77	1.58	4.57	0.09	<.001
Assaults per month per 1,000 (April 2010 to March 2012)	0.38	0.06	1.69	0.23	1.78	5.45	0.10	<.001
Assaults on women per month per 1,000 (April 2010 to March 2012)	0.12	0.01	0.38	0.06	0.94	0.84	0.09	<.001
Male life expectancy at birth 2009–2013	79.94	74.15	91.94	2.52	0.46	0.67	0.05	.004
Female life expectancy at birth 2009–2013	84.29	78.22	92.89	2.28	0.23	0.18	0.03	.200

There was a strong positive association between the incidence of violent crimes recorded by the Metropolitan Police and total assaults recorded by the LAS (*r* = .916; *n* = 533; *p* < .001). Moreover, as shown in Table 2 across the 533 electoral wards there were negative associations between mean income per person and all measures of assault and violent crime incidence rates. In contrast, there were positive associations between inequality (Ward LSOA Gini) and all measures of assault and violent crime incidence rates. Moreover, assault and violent crime incidence rates were negatively associated with both male and female life expectancy.

**Table 2.** Bivariate Associations (Pearson’s Correlation Coefficients) Between Assault (London Ambulance Service) and Violent Crime (Metropolitan Police Service) Incidence Rates (April 2010 to March 2012) and Mean Income per Person (2011), Ward LSOA Gini (2011), and Life Expectancy for Males and Female (2009–2013) for 533 Electoral Wards in London (All Variables Log Transformed).

Data Source	Ward Level Variable	Mean Income Per Person (2011)	Ward LSOA Gini (2011)	Male Life Expectancy at Birth (2009–2013)	Female Life Expectancy at Birth (2009–2013)
Metropolitan Police Service	Violent crimes per 1,000 population per month	−.249**	.239**	−.572**	−.398**
London Ambulance Service	Total assaults per 1,000 population per month	−.262**	.237**	−.566**	−.419**
	Assaults on women per 1,000 population per month	−.227**	.236**	−.523**	−.391**

*Note.* LSOA = lower super output area.

** *p* < .001.

Linear regression analyses were conducted to examine which socioeconomic and demographic variables were the best predictors of the assault and violent crime incidence rates when considered simultaneously (Tables 3–5). Only inequality (Ward LSOA Gini) and male life expectancy at birth were significant predictors of all measures of interpersonal violence (police recorded violent crime, ambulance service recorded total assaults and assaults on women). However, for assaults on women, mean income was an additional predictor.

**Table 3.** Results of Linear Regression Analysis with Socioeconomic and Demographic Variables as Predictors of Metropolitan Police Recorded Violent Crimes per Month per 1,000 Population (April 2010 to March 2012) for 533 Electoral Wards in London (All Variables Log Transformed).

Ward Level Variable	Unstandardized Coefficients		Standardized Coefficients		*p*
	*B*	SE	β	*t*	
Mean income per person	−0.05	0.06	−0.03	−0.79	.431
Ward LSOA Gini	0.20	0.04	0.21	5.59	<.001
Male life expectancy at birth	−8.70	0.84	−0.54	−10.42	<.001
Female life expectancy at birth	0.10	0.91	0.01	0.11	.909

*Note.* Constant = 16.93, model fit *R* = .61, *F*(4,528) =  76.34, *p* < .001. LSOA = lower super output area.

**Table 4.** Results of Linear Regression Analysis with Socioeconomic and Demographic Variables as Predictors of London Ambulance Service Recorded Assaults per Month per 1,000 Population (April 2010 to March 2012) for 533 Electoral Wards in London (All Variables Log Transformed).

Ward Level Variable	Unstandardized Coefficients		Standardized Coefficients		*p*
	*B*	SE	β	*t*	
Mean income per person	−0.09	0.07	−0.05	−1.20	.230
Ward LSOA Gini	0.24	0.04	0.21	5.70	<.001
Male life expectancy at birth	−9.32	0.99	−0.49	−9.45	<.001
Female life expectancy at birth	−0.92	1.08	−0.04	−0.86	.392

*Note.* Constant = 19.69, model fit *R* = .60, *F*(4,528) = 74.75, *p* < .001. LSOA = lower super output area.

**Table 5.** Results of Linear Regression Analysis with Socioeconomic and Demographic Variables as Predictors of London Ambulance Service Recorded Assaults on Females per 1,000 Population (April 2010 to March 2012) for 533 Electoral Wards in London (All Variables Log Transformed).

Ward Level Variable	Unstandardized Coefficients		Standardized Coefficients		*p*
	*B*	SE	β	*t*	
Mean income per person	−0.16	0.07	−0.10	−2.43	.016
Ward LSOA Gini	0.22	0.04	0.20	5.60	<.001
Male life expectancy at birth	−9.58	0.93	−0.52	−10.29	<.001
Female life expectancy at birth	−0.37	1.02	−0.02	−0.36	.716

*Note.* Constant = 18.92, model fit *R* = .63, *F*(4,528) = 87.23, *p* < .001. LSOA = lower super output area.

Collinearity diagnostics for these models indicated that multicollinearity was not a problem with variance inflation factor (VIF) values for the four predictors ranging from 1.13 to 2.27 and tolerance values from 0.44 to 0.88. Visual inspection of P-P plots and residual versus predicted plots indicated that the assumption of homoscedasticity was not violated. Moreover, simple bivariate Pearson’s correlation tests (on log transformed variables) revealed that while there was a small positive association between the inequality measure and mean income (*r* = .25, *n* = 533, *p* < .001), inequality was not associated with either male (*r* = −.08, *n* = 533, *p* = .077) or female (*r* = −.03, *n* = 533, *p* = .495) life expectancy at birth. There were, however, moderate correlations between mean income and both male (*r* = −.50, *n* = 533, *p* < .001) and female (*r* = .43, *n* = 533, *p* < .001) life expectancy.

Ward population sizes did vary across London (Mean 12892.8, *SD* 2365.8), so the regression analyses were each repeated, using a weighted least squares method, weighting for ward population (from the 2011 census). Results were broadly the same, and these are shown in Supplemental Tables 1 to 3. As before, Ward LSOA Gini and male expectancy were significant predictors of all measures of interpersonal violence. However, in these analyses mean income was an additional predictor for the total assault rate and not just assaults on women. As an additional step, robust regression (SPSS 29.0, IBM Corp, Armonk, NY, USA) was used to test whether the results would be similar when testing is carried on the raw (untransformed) variables—and without the exclusion of a ward as an outlier with respect to rates of violence. These regression results are shown in the Supplemental Tables 4 to 6 and again in each case Ward LSOA Gini and male expectancy were the strongest predictors of police recorded violent crime, ambulance service recorded total assaults and assaults on women.

## Discussion

This study provides evidence that a measure of socioeconomic inequality is a better predictor of variation in the incidence of interpersonal violence across London neighborhoods than is a general measure of affluence (mean income). This general pattern was found across both data sources (i.e., Metropolitan Police service violent crime and LAS assault measures) and using different analysis methods. These findings are consistent with previous studies that have examined variation in homicide rates across small scale geographical areas (Szwarcwald et al., 1999; Wilson & Daly, 1997) and make an important additional contribution to our understanding of the role of socioeconomic inequality in interpersonal violence given they are based on a much larger set of incidents consisting mostly of non-lethal events.

Male life expectancy within neighborhoods was found to be an additional important predictor of the incidence of interpersonal violence, similar to Wilson & Daly’s (1997) Chicago study (although that study did not use sex-specific mortality data). Both these findings are consistent with the idea that individuals with poor future prospects will be more willing to engage in risky forms of social competition, as an adaptive response to life circumstances which mean they are less likely to survive to derive benefits from delayed rewards (Daly & Wilson, 2005; Wilson et al., 2009).

As noted in the introduction, associations between levels of inequality and the incidence of interpersonal violence are predicted by both evolutionary psychological perspectives (Daly, 2016) and sociological strain theory (Rennó Santos et al., 2018). Moreover, such associations are also consistent with Hobfoll’s (1989) Conservation of Resources (COR) theory, which suggests that individuals are motivated to acquire and retain resources over their lifespan and experience psychological stress in the face of actual or potential losses (Walt & Jason, 2017). Similar to the sociological strain theory and evolutionary perspective described, the COR also predicts that those with fewer resources (who are potentially most vulnerable to further losses) will employ high risk strategies in an attempt to gain short term pay-offs (Hobfoll, 1989). However, these different perspectives should not be seen as competing explanations—rather different “levels of analysis” in criminology (Durrant & Ward, 2015) with evolutionary approaches focusing on ultimate, and sociological/psychological explanations focusing on proximate, causation.

A strength of this study is that its findings have been demonstrated using two independent datasets—namely police reports of violent crime offences, and ambulance service reports of assaults. As noted by Sutherland et al. (2021) ambulance service data can add important additional information on the incidence of interpersonal violence to supplement data from police records. Both data sources include geographic information—localizing a violent crime or ambulance call-out for an assault incident, to a particular ward. Consequently, the similar findings reported here for analyses based on these different datasets provide convergent evidence on differences in the rates of interpersonal violence between wards.

The nature of the inequality measure used in the present study is rather different to those used in most previous research on interpersonal violence. We did not have access to the individual level income data for small areas that would have been needed to calculate metrics (e.g., Gini coefficients) for inequality between individuals. Instead, aggregate income data for small areas (LSOAs) has been used to calculate a measure of inequality between these small areas within a larger geographical area (a ward—typically containing six to nine LSOAs). Consequently, it is a measure of inequality between neighborhoods within a larger collection of neighborhoods. Arguably a neighborhood measure of inequality may in some ways be more closely linked to psychologically salient experiences of living in an inequitable environment than some individual level measures. Where there are substantial differences in average income between LSOAs within a ward—this will tend to create clusters of relative affluence and clusters of relative poverty which are possibly more conspicuous to people living there than would be scattered individual level differences in income. Indeed, Ramos et al. (2013) hypothesized, that for deprived neighborhoods, the proximity and salience of adjacent more affluent neighborhoods may be an important contributor to experiences of relative deprivation. As a setting for this research, London is a city where such juxtapositions are likely to be particularly salient. It is a city with extreme economic disparities—with inequality levels that are higher than other U.K. regions when considering income after housing costs (The Institute for Fiscal Studies, 2020). Moreover, it is a city where economic inequality is intwined with other forms in inequity as it has a substantially more diverse population than the United Kingdom as a whole. For example, in 2011, London had the highest proportion (40.2%) of minority ethnic groups (Office for National Statistics, 2012).

Nevertheless, there are some significant limitations that arise from the data sources used here. First, we are not able to calculate rates of violent crime and assault perpetration separately for males and females at ward level given the available datasets. This limits the extent to which the findings can be used to assess specific hypotheses about the effects of socioeconomic conditions on interpersonal violence perpetrated by males. However, it is well-established that the majority of the acts of interpersonal violence that come to the attention of the police are perpetrated by males, often young males. For example, the British Crime Survey (BCS) for 2009/2010 found that for violent incidents 84% involved just male offenders while only 9% involved just female offenders (Flatley et al., 2010). Moreover, the BCS found that at that time approximately half (53%) of violent incidents involved offenders aged 16 to 24 years. Young males are also more frequently victims of such incidents, for example, a recent study of patients <18 years old admitted to a U.K. Major Trauma Center with knife wounds (2016–2022) found that 96% were male (Reilly et al., 2023).

A second important limitation is that the boundaries of administrative wards may not map especially well onto the areas which function as neighborhoods from a psychological and behavioral point of view (e.g., where people spend their time). However, for historical reasons the correspondence between administrative wards and behavioral neighborhoods in London is likely greater than might be the case in some other cities and geographical regions. Unlike many planned cities across the world, Greater London in large part, was formed through the expansion and coalescence of small villages and towns. Indeed, it has been described as a “city of villages” (Abercrombie, 1944). Wards are frequently centered on the high streets that once were at the centre of these villages, and now provide foci for shopping, commerce, recreation, and social activities in modern London. Commuting of course complicates matters. People may either gain their experiences of inequity, or participate in acts of violence, anywhere in the city that they travel to, and from exposure to cues in inequality via mainstream and social media. Notwithstanding this, the present study shows that levels of interpersonal violence are higher in neighborhoods where people are likely to experience greater levels of socioeconomic inequality. So whether this violence is being perpetrated by people who live there, or people who travel to spend time there, it has taken place where the perpetrators may be exposed to cues to inequality.

Regression models, in which multiple potential socioeconomic and demographic predictors are entered simultaneously, have been widely used to identify variables that might best account for variation in the incidence of interpersonal violence across geographical areas. However, there are problems with this approach that can arise due to collinearity among predictors and bidirectional relationships between predictors and outcome variables, some of which may have time lags of varying duration (Daly, 2023). As explained above, in this study one bidirectionality issue (e.g., homicide as a cause of lowered life expectancy) is not a major concern as we have used measures of events that are mostly non-lethal assaults rather than homicides, since interpersonal violence is a very infrequent cause of mortality in a U.K. setting. Regarding collinearity—our set of predictors did not violate assumptions regarding multicollinearity when judged by conventional standards (Miles, 2005) with all VIF <4 and tolerance >0.25, and there was only a small correlation between the measure of inequality and mean income, and no association between the inequality measure and the life expectancy measures.

Ideally future research should use longitudinal methods to investigate lagged effects as people may form their sense of societal inequities cumulatively over a number of years (Daly, 2023). However, unfortunately it was not possible to examine such effects using the current data sources. LAS data on assaults has only been published at LSOA/Ward level for the period 2009 to 2015 and the timing of the U.K. census (every 10 years) together with boundary changes mean that it is only possible to reconcile socioeconomic, demographic, and interpersonal violence data for a relatively short time window close to the 2011 census. Moreover, individual level data are ideally needed to address questions about lagged effects. Patterns of mobility mean that even if longitudinal data were available—for small scale geographical areas (e.g., LSOAs and Wards) it is likely that many adults live (and potentially engage in acts of interpersonal violence) in different locations to those where they acquired experiences of socioeconomic inequality during key phases of development.

The findings of strong negative associations between male life expectancy and rates of both police recorded violent crime and ambulance service recorded assaults are consistent with the findings of Wilson & Daly (1997) for Chicago. It is particularly interesting that male, but not female, life expectancy predicted levels of interpersonal violence given the theoretical framework Wilson & Daly (1997) advanced to account for their findings. Briefly, this framework suggests that poor future prospects may encourage individuals to discount the future more heavily and take more extreme, potentially lethal, risks in social competition (Daly & Wilson, 2005; Wilson & Daly, 1997). A life history perspective suggests that this sort of extreme risk-taking and future discounting is more likely to be profitable for males than for females (Hill et al., 1997). Consequently, it should perhaps not be surprising that male future prospects are a better predictor, than female future prospects, of levels of interpersonal violence in the present study (assuming that the majority of the incidents recorded in the data were perpetrated by males).

Overall, the results of the current study provide further evidence of an association between levels of economic inequality and rates of interpersonal violence. The focus on variation in the incidence of non-lethal violence across small geographical areas (neighborhoods), builds on previous research which has largely focused on lethal violence (i.e., homicide) and examined variation between cities, states, provinces, and countries. The inclusion of non-lethal incidents from two independent data sources means the dataset captures a broader range of interpersonal conflict than would be represented in homicide statistics. Moreover, the focus on variation in neighborhood level inequality is important, as variation at this level is likely more closely associated with individual level experiences of inequality than aggregate measures across larger geographical areas would be. Future research investigating individual-level experiences of inequality (particularly for males) is needed to uncover which experiences are most salient for individuals, in order to establish which aspects of inequality have the potential to be generating the most societal harms. To be effective, policy changes and community interventions aimed at ameliorating the negative effects of inequality, need to be informed by an understanding of the psychological processes that mediate the link between inequality in a society, and the behavior of individuals.

## Supplemental Material

sj-docx-1-jiv-10.1177_08862605241271379 – Supplemental material for Economic Inequality, Life Expectancy, and Interpersonal Violence in London Neighborhoods Supplemental material, sj-docx-1-jiv-10.1177_08862605241271379 for Economic Inequality, Life Expectancy, and Interpersonal Violence in London Neighborhoods by Jaye Lee McLaughlin and Nicholas Pound in Journal of Interpersonal Violence

## Notes

1. Future discounting can be defined as “... the rate at which one discounts the future; that is, the rate at which the subjective value of future consumption diminishes relative to the alternative of present consumption (or, if you like, the interest rate required to motivate foregoing consumption)” (Wilson et al., 2009, p. 2835).

2.
https://data.london.gov.uk/download/ward-profiles-and-atlas/5a367ec8-3bdb-4216-9f75-8368a00fe6a2/ward-atlas-data.xls

3.
https://www.met.police.uk/sd/stats-and-data/met/crime-type-definitions/

4.
https://data.ubdc.ac.uk/datasets/monthly-ambulance-service-incidents-lsoa
