# Supplementary material for: Economic Inequality, Life Expectancy, and Interpersonal Violence in London Neighborhoods
Source: J Interpers Violence. 2024 Aug 24;40(13-14):3231–50. doi: 10.1177/08862605241271379 (PMC12130589; doi:10.1177/08862605241271379)
Supplement: sj-docx-1-jiv-10.1177_08862605241271379 – Supplemental material for Economic Inequality, Life Expectancy, and Interpersonal Violence in London Neighborhoods [file sj-docx-1-jiv-10.1177_08862605241271379.docx]

**Supplementary Material**

**Economic inequality, life expectancy and interpersonal violence in London neighbourhoods**

*Journal of Interpersonal Violence*

Jaye Lee McLaughlin^1^ & Nicholas Pound^2^

Centre for Culture & Evolution and Division of Psychology; Department of Life Sciences; College of Health, Medicine & Life Sciences; Brunel University London.

^1^ Email [jaye.mclaughlin@alumni.brunel.ac.uk](mailto:jaye.mclaughlin@alumni.brunel.ac.uk)

^2^ Email: [nicholas.pound@brunel.ac.uk](mailto:nicholas.pound@brunel.ac.uk)

| Supplementary Figure 1: Quintiles for ward mean income per person per year^[[1]](#footnote-1)^ (2011) for 534 wards^[[2]](#footnote-2)^ (90 wards excluded due to LSOA boundary changes shown in grey). |
| --- |
| \| 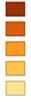 \| > £24,543 \| \| --- \| --- \| \| £19,879 to < £24,543 \| \| £17,159 to < £19,879 \| \| £14,123 to < £17,159 \| \| < £14,123 \|   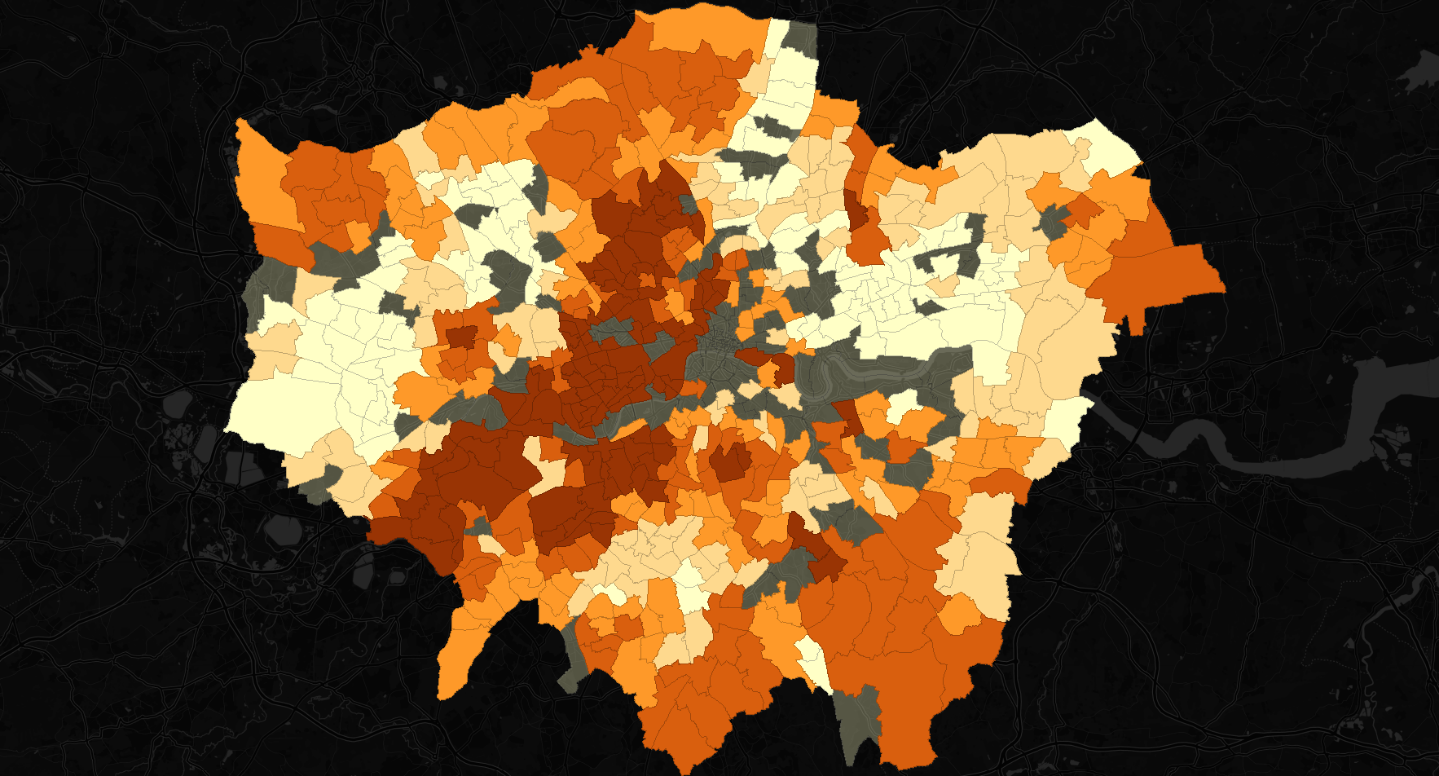 |

| Supplementary Figure 2: Quintiles for ward GINI (2011) for 534 wards (90 wards excluded due to LSOA boundary changes shown in grey). |
| --- |
| \| 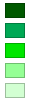 \| > 0.088 \| \| --- \| --- \| \| 0.065 to < 0.088 \| \| 0.051 to < 0.065 \| \| 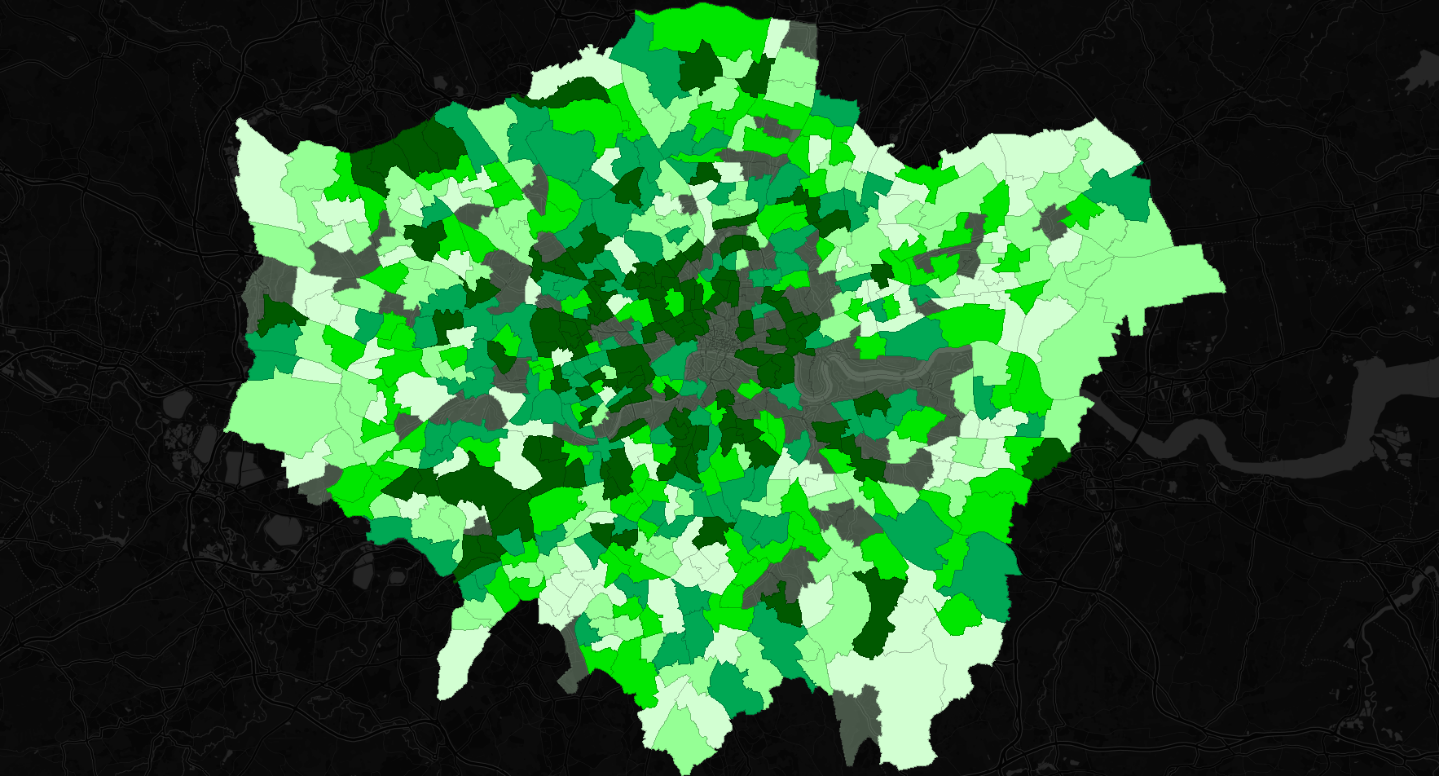0.038 to < 0.051 \| \| < 0.038 \| |
| Supplementary Figure 3: Quintiles for Metropolitan Police recorded violent crimes per month per 1000 population (Apr 2010-Mar 2012) for 534 wards (90 wards excluded due to LSOA boundary changes shown in grey). |
| \| 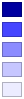 \| > 2.000 \| \| --- \| --- \| \| 1.512 to < 2.000 \| \| 1.191 to < 1.512 \| \| 0.825 to < 1.191 \| \| < 0.825 \|   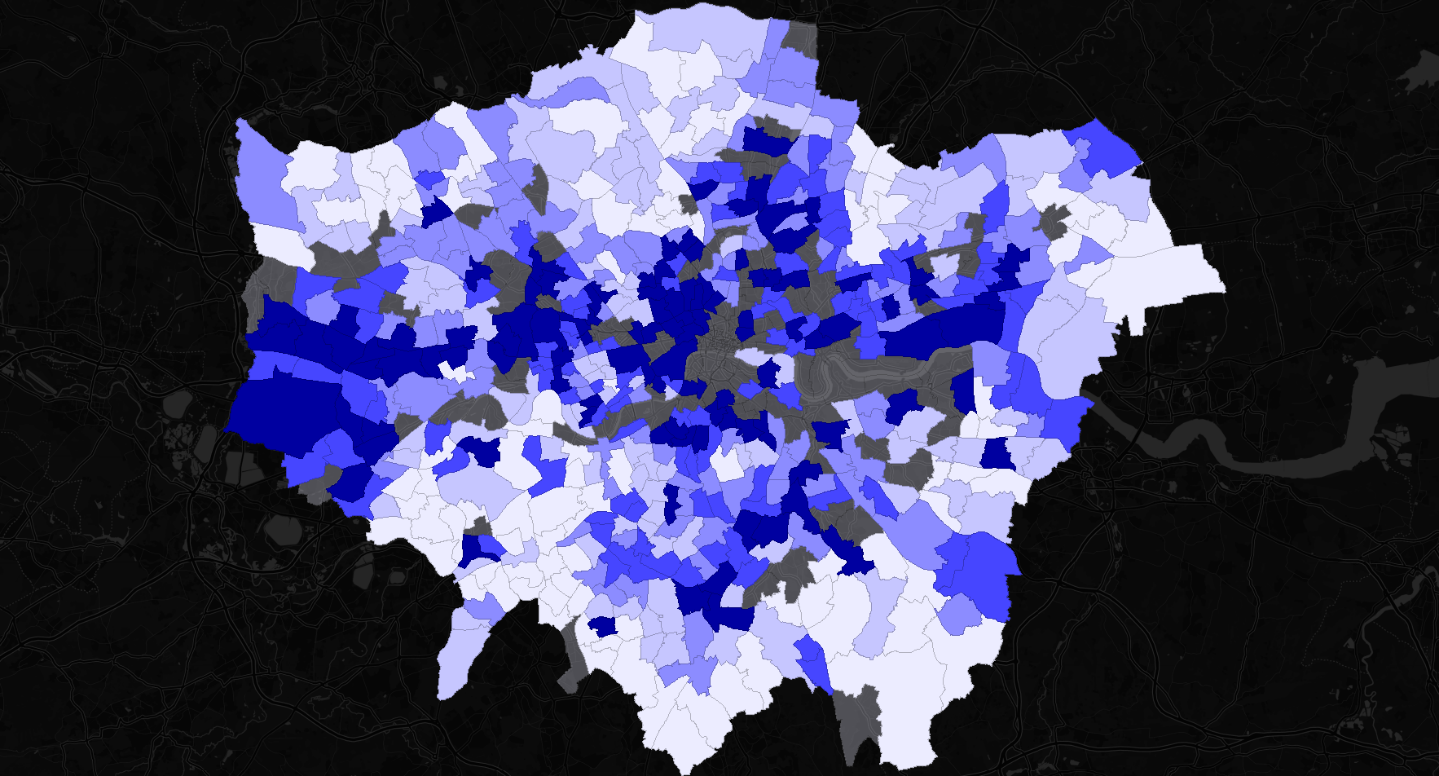 |

| Supplementary Figure 4: Quintiles for London Ambulance Service recorded assaults per month per 1000 population (Apr 2010-Mar 2012) for 534 wards (90 wards excluded due to LSOA boundary changes shown in grey). |
| --- |
| \| 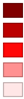 \| > 0.540 \| \| --- \| --- \| \| 0.387 to < 0.540 \| \| 0.276 to < 0.387 \| \| 0.187 to < 0.276 \| \| < 0.187 \|   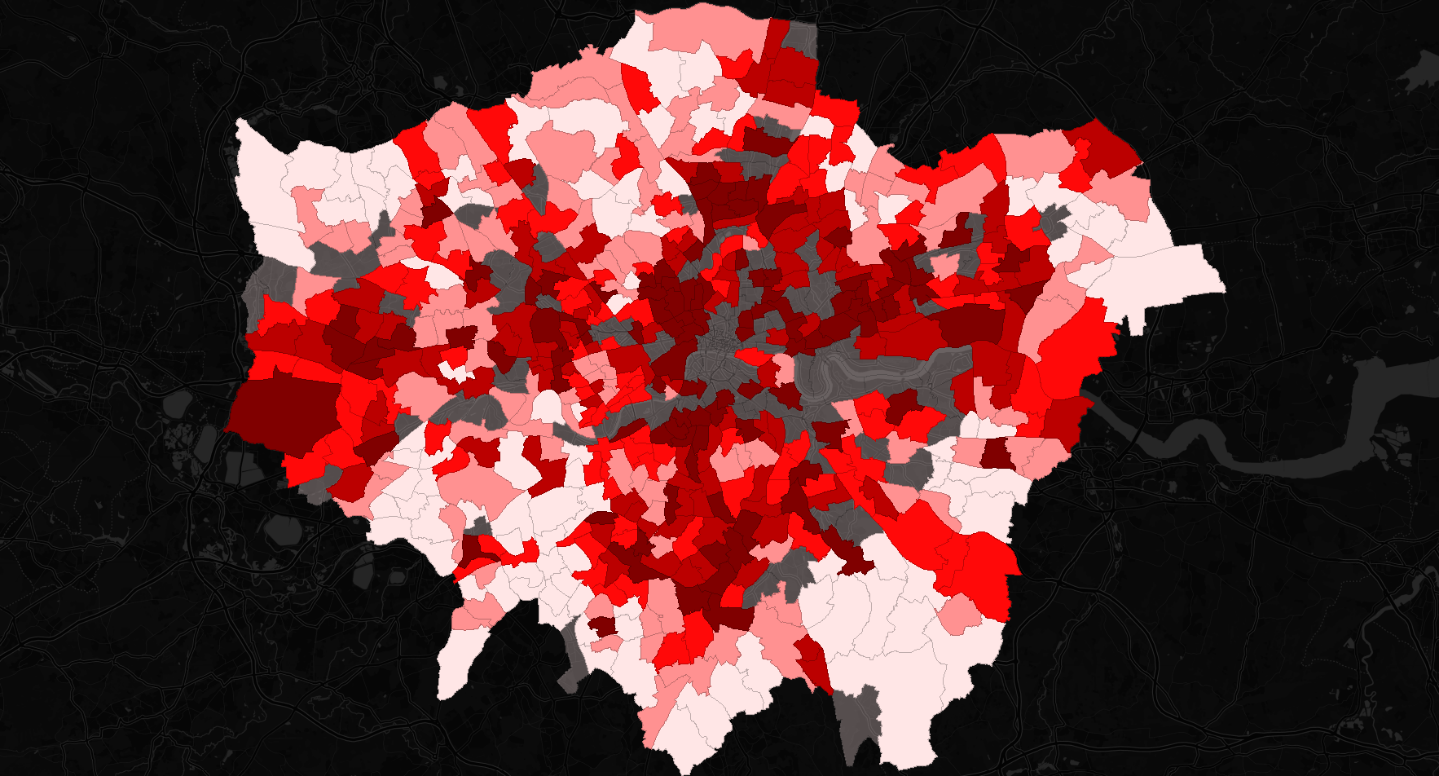 |

#### Supplementary Figure 5

#### Lorenz curve to illustrate calculation of Gini coefficient for one ward (St. Katharine's and Wapping E05000585) consisting of 7 LSOAs.

####
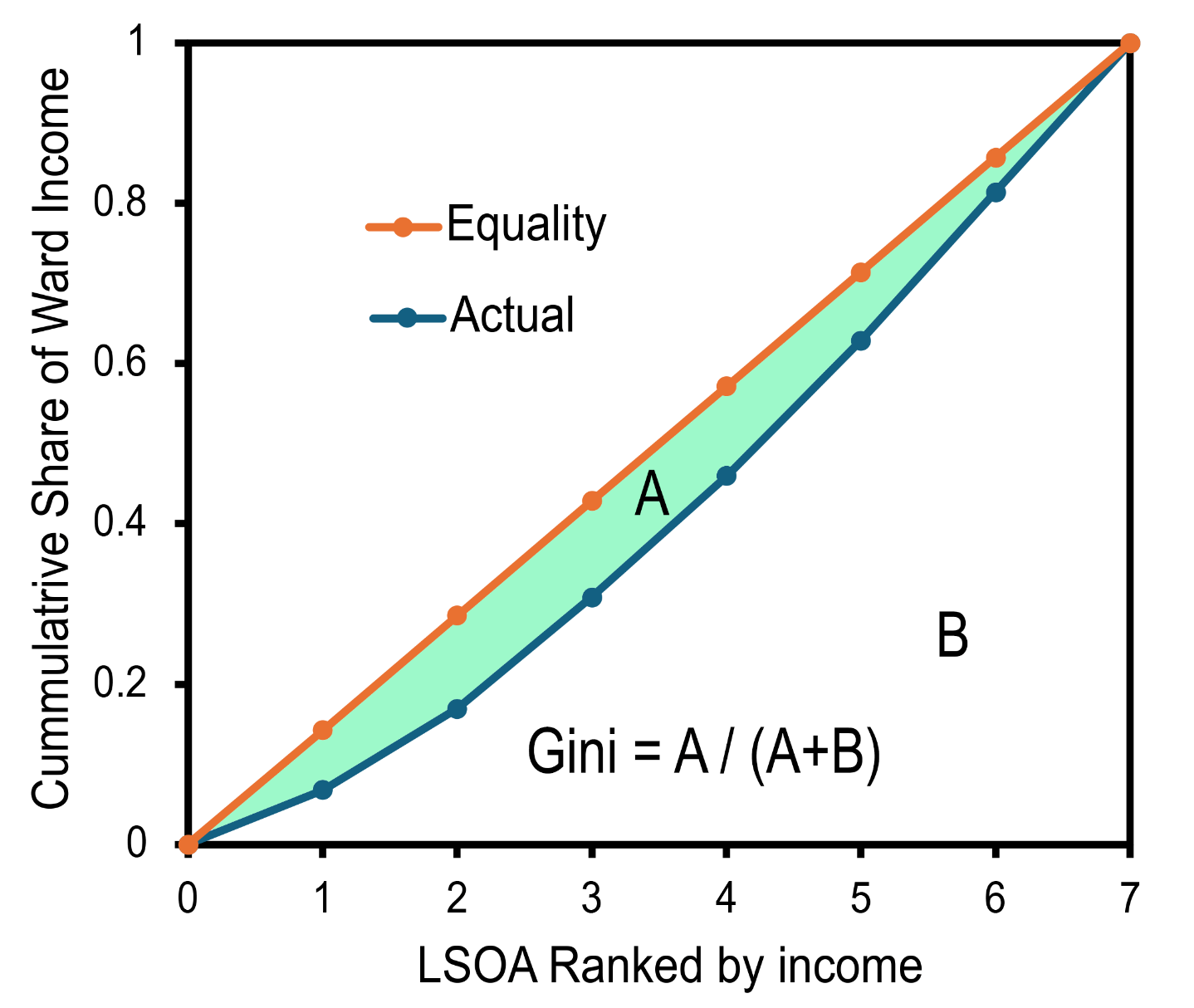


#### Supplementary Table 1

#### Results of weighted least-squares (WLS) linear regression analysis with socioeconomic and demographic variables as predictors of Metropolitan Police recorded violent crimes per month per 1000 population (Apr 2010-Mar 2012). All variables log-transformed. Weighted by ward population (2011 Census).

|  | Unstandardized Coefficients | | Standardized Coefficients |  |  |
| --- | --- | --- | --- | --- | --- |
|  | B | SE | Beta (β) | *t* | *p* |
| Mean income per person | -0.11 | 0.06 | -0.08 | -1.86 | .063 |
| Ward LSOA Gini | 0.19 | 0.04 | 0.20 | 5.36 | <.001 |
| Male life expectancy at birth | -8.73 | 0.84 | -0.54 | -10.42 | <.001 |
| Female life expectancy at birth | 0.41 | 0.91 | 0.02 | 0.45 | .652 |
| Note. Constant = 16.66, Model Fit *R* = .61, *F*(4,528) = 77.49, *p* < .001 | | | | | |

#### Supplementary Table 2

#### Results of weighted least-squares (WLS) linear regression analysis with socioeconomic and demographic variables as predictors of London Ambulance Service recorded assaults per month per 1000 population (Apr 2010-Mar 2012). All variables log-transformed. Weighted by ward population (2011 Census).

|  | Unstandardized Coefficients | | Standardized Coefficients |  |  |
| --- | --- | --- | --- | --- | --- |
|  | B | SE | Beta (β) | *t* | *p* |
| Mean income per person | -0.17 | 0.07 | -0.10 | -2.38 | .018 |
| Ward LSOA Gini | 0.23 | 0.04 | 0.20 | 5.43 | <.001 |
| Male life expectancy at birth | -9.40 | 1.00 | -0.49 | -9.42 | <.001 |
| Female life expectancy at birth | -0.66 | 1.09 | -0.03 | -0.60 | .546 |
| Note. Constant = 19.68, Model Fit *R* = .61, *F*(4,528) = 76.85, *p* < .001 | | | | | |

#### Supplementary Table 3

#### Results of weighted least-squares (WLS) linear regression analysis with socioeconomic and demographic variables as predictors of London Ambulance Service recorded assaults on females per month per 1000 population (Apr 2010-Mar 2012). All variables log-transformed. Weighted by population (2011 Census).

|  | Unstandardized Coefficients | | Standardized Coefficients |  |  |
| --- | --- | --- | --- | --- | --- |
|  | B | SE | Beta (β) | *t* | *p* |
| Mean income per person | -0.21 | 0.07 | -0.13 | -3.08 | .002 |
| Ward LSOA Gini | 0.21 | 0.04 | 0.18 | 5.13 | <.001 |
| Male life expectancy at birth | -9.62 | 0.94 | -0.52 | -10.20 | <.001 |
| Female life expectancy at birth | -0.23 | 1.03 | -0.01 | -0.23 | .82 |
| Note. Constant = 18.92, Model Fit *R* = .63, *F*(4,528) = 86.40, *p* < .001 | | | | | |

**Robust linear regression analyses**

Robust linear regression analyses were carried out to test whether the main results would replicate without transformation of the predictor or outcome variables and without exclusion of an outlier due to high violent crime rates. Analyses were carried about using SPSS 29.0 using Robust Regression package version 1.28 – which uses the rlm function from the R MASS package (<https://github.com/IBMPredictiveAnalytics/SPSSINC_ROBUST_REGR>).

#### Supplementary Table 4

#### Results of robust linear regression analysis with socioeconomic and demographic variables as predictors of Metropolitan Police Recorded violent crimes per month per 1000 population (Apr 2010-Mar 2012) across 524 wards, with no transformations applied to predictor or outcome variables or exclusion of outliers.

|  | Value | Std Error | *t* |
| --- | --- | --- | --- |
| Intercept | 13.45 | .888 | 15.14 |
| Mean income per person | 4.67 x 10^-6^ | .000 | 1.64 |
| Ward LSOA Gini | 3.25 | .671 | 4.85 |
| Male life expectancy at birth | -0.15 | .012 | -11.86 |
| Female life expectancy at birth | -0.01 | .013 | -0.60 |
| Residual standard error = 0.444, *df* = 529 | | | |

#### Supplementary Table 5

#### Results of robust linear regression analysis with socioeconomic and demographic variables as predictors of London Ambulance Service recorded assaults per month per 1000 population (Apr 2010-Mar 2012) across 524 wards, with no transformations applied to predictor or outcome variables or exclusion of outliers.

|  | Value | Std Error | *t* |
| --- | --- | --- | --- |
| Intercept | 3.83 | 0.271 | 14.11 |
| Mean income per person | 1.03 x 10^-6^ | 8.70 x 10^-7^ | 1.18 |
| Ward LSOA Gini | 0.96 | 0.205 | 4.66 |
| Male life expectancy at birth | -0.04 | 0.004 | -9.66 |
| Female life expectancy at birth | -0.01 | 0.004 | -1.99 |
| Residual standard error = 0.131, *df* = 529 | | | |

#### Supplementary Table 6

#### Results of robust linear regression analysis with socioeconomic and demographic variables as predictors of London Ambulance Service recorded assaults on females per month per 1000 population (Apr 2010-Mar 2012) across 524 wards, with no transformations applied to predictor or outcome variables or exclusion of outliers.

|  | Value | Std Error | *t* |
| --- | --- | --- | --- |
| Intercept | 1.18 | 0.085 | 13.83 |
| Mean income per person | 5.68 x 10^-8^ | 2.73 x 10^-7^ | 0.21 |
| Ward LSOA Gini | 0.26 | 0.064 | 3.99 |
| Male life expectancy at birth | -0.01 | 0.001 | -11.24 |
| Female life expectancy at birth | 0.00 | 0.001 | -0.18 |
| Residual standard error = 0.042, *df* = 529 | | | |

1. Mean annual income per person = mean household income in ward × number of households in ward / total ward population [↑](#footnote-ref-1)
2. St James (E05000644) was excluded from the analyses as an outlier (violent crime 8.8 x SD above mean) but is shown in the figures above for information purposes. [↑](#footnote-ref-2)
